# Measuring the multidimensional aspects of tolerability

**DOI:** 10.1002/cncr.70085

**Published:** 2025-10-22

**Authors:** Sandra A. Mitchell, Rachel D. Altshuler, Diane C. St. Germain, Brennan Parmelee Streck, Alice P. Chen, Lori M. Minasian

**Affiliations:** ^1^ Division of Cancer Control and Population Sciences National Cancer Institute National Institutes of Health Rockville Maryland USA; ^2^ Division of Cancer Prevention National Cancer Institute National Institutes of Health Rockville Maryland USA; ^3^ Kelly Government Solutions Rockville Maryland USA; ^4^ Division of Cancer Treatment and Diagnosis National Cancer Institute National Institutes of Health Rockville Maryland USA

**Keywords:** cancer treatment, Common Terminology Criteria for Adverse Events (CTCAE), patient‐reported outcomes, Patient‐Reported Outcomes version of the Common Terminology Criteria for Adverse Events (PRO‐CTCAE), tolerability, toxicity

## Abstract

As new cancer therapies emerge and evolve, there is a need for a better understanding of their safety and tolerability. Safety and tolerability are distinct, albeit related, constructs; an unsafe treatment cannot be considered tolerable, whereas safe treatments may not be tolerable to some patients. Cancer treatment tolerability is a multidimensional construct, and is influenced both by the profile of adverse events and by whole‐person factors. This commentary provides definitions of safety, tolerability, adverse events, and toxicity by relating these constructs to the adverse event reporting paradigm. Measures, including summary indicators, that reflect the tolerability of cancer treatments are also identified. The commentary concludes with a discussion of how evaluations of tolerability may be meaningfully incorporated into the design and interpretation of future cancer treatment trials.

## Abstract 2

An understanding of treatment tolerability includes both the profile of adverse events and summary indicators of tolerability, interpreted in the context of whole‐person factors. Tolerability measurements that incorporate both clinician‐ and patient‐reported outcomes support the most complete understanding of the benefits and risks of a cancer treatment, and are essential for shared decision‐making and the provision of optimized supportive care.

## EVOLVING PERSPECTIVES ON CANCER TREATMENT TOLERABILITY

Safety and tolerability are fundamental to drawing conclusions about the effectiveness of cancer therapies, including comparative effectiveness, and complement clinical trial end points such as progression‐free survival, overall survival, and clinical benefit.^1^ All cancer‐directed therapies have the potential to cause toxicities that can result in early treatment discontinuation, dose reductions, or patient nonadherence to therapy and, as a consequence, inferiorities in cancer treatment outcomes^2^, ^3^ and impairments in health‐related quality of life (HRQOL)^4^ and functional status.^5^, ^6^

In this commentary, we outline key concepts to be considered when evaluating cancer treatment tolerability. We begin by offering definitions of tolerability and related concepts such as adverse events (AEs), toxicity, and safety by situating these concepts within the AE monitoring paradigm. This is followed by a summary of direct and indirect approaches to the measurement of tolerability, and a brief consideration of the ways in which whole‐person factors may meaningfully shape our interpretations and conclusions about cancer treatment tolerability, at both the group/trial level and the person/individual level. We conclude by highlighting selected tolerability‐related considerations in cancer treatment trial design.

## GAUGING SAFETY AND TOLERABILITY VIA AE MONITORING

### AEs, toxicity, safety, and tolerability

A comprehensive understanding of tolerability must also incorporate an understanding of related constructs including AEs, toxicity, and safety and the related constructs of dose‐limiting toxicity, serious adverse events (SAEs), and maximum tolerated dose (MTD). Table 1 presents definitions of these key constructs drawn from the pharmacovigilance literature. AEs are harmful, unfavorable occurrences that arise during medical treatment or the conduct of a clinical trial, regardless of whether the event is attributable to the treatment. AEs that are determined to be probably or possibly related to a treatment are considered to represent the toxicities or side effects of that treatment (see Table 1).

**TABLE 1:** Concepts and definitions.

Concept	Definition
Adverse event	Any unexpected, harmful, or unfavorable occurrences, medical conditions, symptoms, or signs that happen during medical treatment or a clinical trial. The occurrence of an AE does not necessarily mean that the event is definitively attributable to the treatment being studied in a trial
Dose‐limiting toxicity	Describes side effects of a treatment that are serious enough to prevent an increase in the dose level of that treatment
Maximum tolerated dose	The highest dose of a treatment that does not cause unacceptable side effects. The maximum tolerated dose is determined in clinical trials by testing increasing doses on different groups of people until the highest dose with acceptable side effects is found
Serious adverse event	A serious adverse event refers to any expected or unexpected AE, related or unrelated to the therapy being studied, occurring at any agent dose, any phase of product or procedure testing, that results in death, a life‐threatening AE, requirement of inpatient hospitalization or prolongation of existing hospitalization, or a persistent or significant disability or incapacity
Safety	Safety is an evaluation process designed to detect, assess, monitor, prevent, and understand AEs experienced by patients receiving a treatment regimen. An evaluation of safety yields an understanding of the risks, harms, and benefits of a treatment, medication, or therapeutic regimen. Safety includes the frequency, seriousness, severity, reversibility, duration, and consequences of observed AEs and reactions
Toxicity	A potentially harmful, untoward, or unpleasant reaction that results from or is associated with an intervention or medicinal product, and that may be (1) associated with a clinical sign or syndrome, (2) reflected in a laboratory test or clinical investigation, and/or (3) experienced by the patient as a sign or symptom
Tolerability	The degree to which AEs resulting from or associated with a treatment affect the ability or desire of the patient to adhere to the planned dose and/or schedule of a regimen or therapy. A complete understanding of tolerability should include direct measurement from the patient of how they are feeling and functioning while receiving the treatment

*Note:* Based on information from Aronson 2023,^7^ Friends of Cancer Research 2025,^8^ National Cancer Institute 2025,^9^ National Center for Advancing Translational Sciences 2025,^142^ Peipert & Smith 2022,^10^ Ray 2018,^11^ Shader 2018,^12^ Singh & Loke 2012,^13^ and Stanulović 2022.^14^

Abbreviation: AE, adverse event.

Safety, with respect to the development and testing of new medical treatments, is an evaluation process designed to detect, assess, monitor, prevent, and understand AEs, including SAEs, experienced by patients receiving a treatment regimen. Tolerability is the degree to which AEs affect the ability or desire of the patient to adhere to the planned dose and/or schedule of a treatment regimen. On the basis of the expectation that chemotherapy agents have a monotonically increasing dose–efficacy relationship, conventional phase 1/2 clinical trial designs have focused on identifying the MTD. Determinations about the recommended phase 2 dose (for use in subsequent trials) are then determined on the basis of the highest dose that will minimize SAEs and cumulative toxicity and provide long‐term tolerability.^15^

However, newer cancer treatment approaches, such as molecularly targeted agents and immunotherapies, are often not dose dependent. Thus, cancer treatment trial designs are emerging that do not escalate to the MTD, in favor of finding the dose or range of doses that optimize the effectiveness of an agent. This paradigm shift has stimulated the use of novel trial designs that identify the dose and/or schedule of a drug that is tolerable without sacrificing efficacy.^16^, ^17^ It has also accelerated the imperative for the inclusion of patient‐centered indicators of treatment tolerability in early‐ and later phase trials of all new investigational cancer therapies.^18^, ^19^, ^20^

Although the terms “safety” and “tolerability” are sometimes used interchangeably, they represent somewhat different concepts.^14^, ^21^ For example, an agent may be considered tolerable, whereas laboratory testing or other evaluations show it to be unsafe (e.g., producing irreversible renal insufficiency). Conversely, a treatment may be safe on the basis of objective clinician assessments, including relevant laboratory or diagnostic studies, and yet that treatment may produce toxicities that are unacceptable to the patient or that warrant dose interruption or discontinuation. Conclusions about safety and tolerability should be based on a systematic and generalizable evaluation of the absence or presence of harms, as determined by relevant questioning, observation, and testing.^12^

Safety and tolerability are considered in terms of the risks and the magnitude of harm, and particularly with respect to tolerability, there is a value judgment of the acceptability of those risks based on their probability of occurring and their effect on patients.^22^ The health condition for which the treatment is being given is also relevant because judgments about acceptable risk depend on the type and severity of the underlying health problem being addressed, the treatment alternatives, and the magnitude of potential benefit. Relevant contextual factors in drawing conclusions about safety and tolerability also include the population (and any inherent subgroup characteristics that might increase susceptibility to adverse effects) and the specific conditions of use (e.g., research settings vs. nonresearch settings).^12^, ^21^, ^23^ This judgment about the acceptability of the risks must be deliberated from the perspectives of patients, clinicians, society, and other appropriate decision makers.^24^, ^25^, ^26^

It is important to note that the concept of tolerability should be considered as both a between‐group and a within‐person construct.^10^, ^27^ Interpretation at the group or trial level allows for generalizable comparisons and conclusions about the aggregated tolerability of a specific treatment regimen being tested in a trial. Within‐person interpretations of treatment tolerability can be applied to inform patient–clinician shared decision‐making and to individualize the provision of toxicity‐directed interventions and self‐management support.^28^, ^29^

### AE‐monitoring paradigm

AE monitoring, severity rating, and reporting are essential components of any clinical trial evaluating a new cancer therapy. AE monitoring is the process of systematically identifying and tracking unfavorable clinical outcomes associated with a treatment regimen to draw conclusions about its safety and tolerability. Therapeutic response to a new cancer therapy is determined on the basis of outcomes such as survival, disease modification, clinicians’ global impression of treatment benefit, and patient‐reported outcomes (PROs) such as HRQOL gathered at periodic prespecified intervals. In contrast, safety and tolerability determinations require continuous surveillance and monitoring for AEs across the course of a trial, together with a standardized approach to categorization and severity rating. Important characteristics to understand about AEs include not only their specific nature (e.g., neuropathy, nausea, acute kidney injury, noninfectious pneumonia, gait disturbance, and ileus) but also their severity, reversibility, consequences, chronicity, and pattern of onset, offset, accumulation, and co‐occurrence.^30^, ^31^ AE monitoring ensures that individual study participants are not harmed by treatment, and allows for generalizable conclusions to be drawn at the trial level regarding a regimen’s safety and tolerability and the anticipated profile of treatment‐related toxicities. A well‐characterized AE profile derived at the trial level also informs individual‐level provision of anticipatory guidance and the delivery of targeted supportive care to mitigate and manage adverse effects and deleterious outcomes.

## MEASURING THE MULTIDIMENSIONAL ASPECTS OF TOLERABILITY

Over the past several years, there has been growing momentum to more systematically incorporate the patient’s perspective into AE reporting and the evaluation of cancer treatment tolerability.^20^, ^32^, ^33^ This has been motivated in part by the greater use of novel immunologic and molecularly targeted agents that are chronically administered by patients in their homes, often over months or years.^34^ These novel agents may also have a more subtle, distinctive, and/or enduring toxicity profile,^35^ and chronic, lower grade but still bothersome toxicities have been implicated in elective treatment discontinuation and patient nonadherence to therapy.^36^, ^37^ A growing consensus that patients can best gauge outcomes such as symptoms, function, and HRQOL has accompanied this trend,^38^, ^39^ together with an expanding interest in using comparative effectiveness methods^40^ and value frameworks^41^, ^42^ to improve the patient centeredness of care and provide tailored, personalized treatment. The definition of tolerability offered in Table 1 emphasizes the patient centeredness of this construct, in which tolerability reflects the extent to which the toxicities and harms associated with a treatment regimen can be accepted by a patient.

In addition to PROs, other indicators of tolerability include rates of Common Terminology Criteria for Adverse Events (CTCAE) grade 2 or 3 AEs, dose reductions/dose interruptions and treatment discontinuation due to AEs, hospitalizations, and elective patient discontinuations due to moderate or severe toxicity. Although tolerability is directly related to the profile of treatment‐related AEs, it is also influenced by patient‐ and system‐level factors, including comorbidities, treatment preferences, physiologic resilience, psychosocial health, health behaviors, age/developmental stage, lifestyle, geographic environment, socioeconomic status, and social support.^10^, ^38^, ^43^ Thus, a full understanding of tolerability requires a comprehensive ascertainment of the profile of toxicities considered in the context of whole‐person factors that may influence an individual patient’s capacity, desire, or willingness to persist with treatment, although not all of these domains will be measured in any given trial.

**FIGURE 1:**
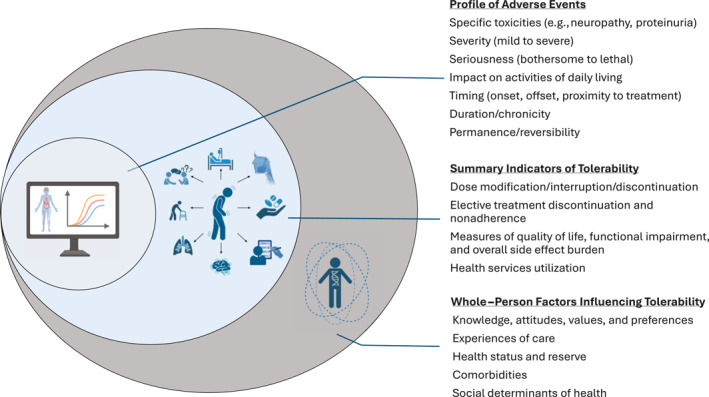
Multidimensional aspects of tolerability.

As shown in Figure 1, this tolerability framework situates the domains articulated by the Food and Drug Administration’s core PROs in cancer trials draft guidance, such as symptomatic AEs, overall side effect impact summary measures, and physical and role functioning,^44^ within the broader AE‐reporting paradigm (which encompasses a comprehensive AE profile as well as summary indicators of tolerability, as discussed below). It serves as a unifying conceptual perspective via which trialists, PRO‐focused investigators, patient advocates, and regulators can consider what tolerability domains to measure and how to simultaneously interpret both clinician‐ and patient‐reported tolerability indicators.

### Profiling of AEs

Monitoring and characterization of AEs are essential aspects of every cancer clinical trial, which ensure that individual study participants are not harmed by treatment and allow for valid, reliable, and generalizable conclusions about a regimen’s safety and tolerability to be made at the trial level. The standard system for defining, classifying, reporting, and monitoring AEs in cancer clinical trials is the National Cancer Institute’s CTCAE, now in version 5.^45^ The CTCAE was designed for evaluating safety. CTCAE toxicities are graded by health care providers on the basis of the clinician’s review of systems, physical examination, and review of data derived from laboratory and diagnostic studies. Clinicians incorporate patient reporting of their experiences into the assignment of CTCAE grades; however, notably, these experiences are filtered through the interpretations of a clinician. The CTCAE provides for standardization and efficiency across studies in the identification and severity grading of AEs, including asymptomatic AEs.

There are several limitations to measuring tolerability via the CTCAE alone. First, although high‐grade AEs (grade 3 [medically significant], grade 4 [life threatening], and grade 5 [death]) have important implications for treatment safety, lower grade AEs (grades 1 and 2) can be underreported yet still affect tolerability.^46^ Second, CTCAE severity grades of symptomatic AEs such as fatigue, pain, nausea, and shortness of breath may not fully correspond to the frequency, severity, or interference scores obtained via direct patient self‐report with PROs.^32^, ^47^ Clinician AE grading also generally occurs in association with clinic visits, and thus AEs that occur between visits may be missed. As a result of these gaps, we may lack a full understanding of the severity, chronicity, and impact of AEs in a clinical trial.^48^, ^49^ This incomplete picture of toxicities hinders the drug development process, and at the individual patient level may limit optimal supportive care to mitigate toxicities and improve tolerability. The systematic assessment of symptomatic AEs via PROs provides additional information about tolerability that is complementary to the assessments captured by clinicians using the CTCAE. The inclusion of patient self‐report in the assessment of AEs also has the potential to improve patient satisfaction and communication with their clinicians and to foster early detection of potentially serious AEs.^50^

To support the inclusion of the patient experience of adverse treatment effects in evaluations of cancer treatment tolerability and to complement the CTCAE, the National Cancer Institute developed and rigorously validated the PRO‐CTCAE measurement system.^51^, ^52^, ^53^, ^54^, ^55^, ^56^ The PRO‐CTCAE is designed to profile the symptomatic AEs of cancer treatment, and has an item structure that allows the ascertainment of the full range of patient‐reportable treatment AEs, including AEs that are not typically represented on other symptom inventories, such as nail dystrophy, photosensitivity, bruising, chills, and fecal incontinence.^57^, ^58^ As with all AE reporting, capturing both expected and unexpected events is critically important. A set of relevant AEs for routine surveillance by clinicians using the CTCAE should be identified in the Comprehensive Adverse Event and Potential Risks (CAEPR) or AE‐monitoring section of the study protocol; other AEs that arise are graded as unexpected events. Similarly, the symptomatic AEs selected should be those likely to be associated with the regimen under study on the basis of the mechanism of action and early clinical data,^32^ or symptoms that might be expected to be present at baseline due to prior cancer treatments and/or comorbid conditions.^59^ Symptomatic toxicities selected for surveillance should match the CAEPR, and in a multiarm trial all participants should report on the same PRO‐CTCAE items across the different trial arms to reduce reporting bias. Additionally, the capture of unsolicited symptomatic AEs via a write‐in or verbatim text feature helps to minimize possible biases in item selection, and may identify early signals of previously unrecognized AEs.^60^, ^61^

The assessment schedule and intervals for symptomatic AE assessment with PROs must be appropriate for the regimen, patient population, and expected trajectory of AE onset and duration. Frequency and timing of patient reporting may vary on the basis of the specific trial design; however, there is evidence that sparse assessment intervals, particularly when coupled with a 24‐h recall period, may result in inaccuracies in symptomatic AE detection.^62^, ^63^ In all trials, a baseline assessment is essential to evaluate the prevalence and severity of symptoms before the study‐specific intervention is administered. Baseline values have interpretive value in AE attribution by examining group imbalance in multiarm trials, by gauging exacerbation in established toxicities caused by prior therapies, and as a covariate in longitudinal models. In some trial contexts, symptomatic AEs may also be captured via PROs after treatment discontinuation.^64^ The study design and analysis plan should incorporate published guidelines for studies that include a PRO end point.^65^, ^66^

PRO‐CTCAE scores are intended for the descriptive reporting of symptomatic AEs and their direct effects on functioning in daily life. Clinician grading with the CTCAE remains the basis for determining protocol eligibility, dose modifications or interruptions, treatment discontinuation, identification of dose‐limiting AEs, and safety reporting to regulatory agencies.^67^ The integration of both clinician‐ and patient‐reported measures supports a comprehensive understanding of treatment tolerability.

### Summary indicators of treatment tolerability

Although a precise and accurate profiling of AEs via the CTCAE and PRO‐CTCAE is essential, other indicators can quantitatively summarize the extent and clinical impact of AEs, and thereby contribute additional insights into treatment tolerability. Such summary indicators include dose preservation, adherence and persistence,^68^ health services utilization, self‐reported measures of HRQOL and physical functioning,^69^, ^70^ single‐item measures of overall side effect burden,^71^ performance‐based and instrumented measures such as the 6‐min walk,^72^ and actigraphy.^73^

Hospitalizations, unscheduled health care visits, the inability to receive the full dose and duration of a planned treatment, elective treatment discontinuation, and nonadherence are all indirect indicators of treatment tolerability.^14^, ^36^, ^74^ However, the interpretation of these metrics as indicators of tolerability also requires that investigators address confounding introduced by chronic conditions^75^, ^76^ and the side effects of noncancer medications.^77^ Measures of HRQOL and well‐being such as the Functional Assessment of Cancer Therapy–General (FACT‐G), European Organisation for Research and Treatment of Cancer (EORTC) Core Quality of Life Questionnaire C30, and self‐reported health status, physical function, and activities of daily living^38^ are particularly helpful in gauging the cumulative and whole‐person impact of multiple symptomatic AEs. Measures of HRQOL used in conjunction with the PRO‐CTCAE may yield additional insights into the impact of AEs, especially those that are low grade or may exert differential impacts at the individual level among those with organ system compromise, limited functional reserve, multimorbidity, and unmet social needs. Patient‐reported physical function may be a particularly important summary indicator of treatment tolerability, particularly in older adults.^78^, ^79^

There is evidence that single‐item measures of overall side effect burden—such as FACT GP5 (“I am bothered by the side effects of treatment”) and EORTC Q168 (“To what extent have you been troubled with side effects from your treatment?”)—and indicators of the patient’s willingness to stay on treatment even while enduring side effects—such as the Patient Acceptable Symptom State^80^—are valid and reliable, and may have predictive value and utility for stratification and subgroup analyses.^81^, ^82^, ^83^, ^84^ Strengths of single‐item summary measures of side effect burden include their brevity, face validity, and ease of interpretation. However, as with many single‐item measures, they may demonstrate insensitivity to between‐person differences, ceiling effects, and nonresponsiveness to change, particularly in the setting of missing assessments.^85^

### Whole‐person factors influencing treatment tolerability

There is evidence that patient preferences,^26^ health status and functional reserve,^79^, ^86^ experiences of care,^87^ and social determinants of health such as social support,^88^ poverty,^89^ or rurality^90^ may directly or indirectly influence experiences of tolerability. For example, two patients who have been prescribed the same regimen may have differing experiences of tolerability on the basis of their specific circumstances. This might be because one patient has pain from a spine injury that occurred 10 years ago, has a stressful job with a supervisor who does not support the need for periodic absence to attend clinic appointments, and is a sole provider for two young children with little community support, whereas another patient has no chronic conditions, is not currently working, and has a strong support system and abundant financial resources. Although both patients may experience a similar AE profile, their overall experience of tolerability may be quite different. There is empirical evidence of individual differences in tolerability based on socioeconomic, environmental, cultural, psychosocial, and behavioral factors.^87^, ^91^, ^92^, ^93^, ^94^, ^95^, ^96^ Evidence also points to several whole‐person factors that place patients at risk for more frequent AEs,^92^ greater symptom burden,^97^, ^98^ and inferiorities in HRQOL during and after cancer treatment.^99^, ^100^ Financial hardship, deficiencies in patient–provider communication and rapport, low health literacy, comorbid conditions, and barriers to accessing needed care can all influence tolerability and adversely affect effective self‐management.^101^, ^102^ These whole‐person factors can be targeted with proactive, multilevel, and interdisciplinary interventions designed to improve treatment tolerability, preserve dose density, and optimize recovery and overall well‐being.^103^, ^104^, ^105^, ^106^

## TOLERABILITY MEASUREMENT CONSIDERATIONS IN CANCER TREATMENT TRIAL DESIGN

A thoroughly characterized AE profile is especially important in phase 1 trials, where AEs are the primary end point and sample sizes may be small. It is similarly important in phase 2 trials, in which the goal is to gauge preliminary signals about relative benefits versus risk. In early‐phase trials, the inclusion of both the PRO‐CTCAE and summary indicators of tolerability sets the stage for more focused and efficient tolerability evaluations in later phase trials, and may help to define the supportive care that should be included as part of the standard of care in subsequent trials.^107^ The PRO‐CTCAE and summary indicators of tolerability may also have interpretive value in the context of dose‐optimizing study designs^108^, ^109^ by helping to define candidate dose ranges/schedules to be studied in the expansion cohort and confirming improved tolerability in dose comparison designs.^18^, ^110^, ^111^, ^112^

In later phases of therapy development, the emphasis in understanding tolerability shifts from defining the MTD or exploring the AE profile associated with a range of potentially efficacious dose levels to evaluating combination or sequential multiagent regimens^113^ or comparing two or more effective treatment approaches in larger samples.^114^ Patient‐reported AEs supplemented by summary indicators of tolerability may also provide a sensitive secondary end point in comparative effectiveness studies or studies of new approaches to supportive cancer care where toxicity reduction and improving tolerability is a goal of treatment.^95^

Precise characterization of the profiles of clinician‐ and patient‐reported AEs, including low‐grade AEs, may be especially important in prevention, adjuvant, and maintenance therapy trials. Moreover, with many of these therapies, patients’ adherence and persistence over time is fundamental to treatment effectiveness. As such, comparatively mild toxicities, especially experienced over an extended period, may contribute to nonadherence or lead patients to request early treatment discontinuation.^68^

Systematically characterizing baseline symptoms at study entry complements baseline assessments of vital organ functioning, and can result in a more accurate and efficient attribution of symptomatic AEs^115^ and inform prediction of favorable^116^ and unfavorable outcomes.^117^ The trajectory of symptomatic AEs may also provide additional information about the duration^118^ and chronicity^119^, ^120^ of toxicities. Symptomatic AEs may also be important covariates in analyses to understand change over time (both improvements and worsening) in HRQOL outcomes. Identifying subgroups experiencing distinct toxicity profiles on the basis of the awareness that whole‐person factors influence treatment tolerability can also be informative for hypothesis generation about the mechanisms underlying specific AEs. This knowledge can be integrated with biomarker and omic profiles to improve AE risk prediction.^121^ Patient self‐report of symptomatic AEs may be particularly useful in identifying subgroups who are at greatest risk for adverse treatment outcomes, and would therefore benefit from tailored supportive care management.

Despite the importance of understanding cancer treatment tolerability, challenges in capturing, analyzing, and reporting symptomatic AE data have been documented.^122^, ^123^ A range of resources and methodological tools exists to support early‐phase trial study teams in implementing best practices during trial design,^124^ protocol development,^125^ PRO data collection and study implementation,^65^, ^126^, ^127^ and analysis and interpretation.^128^ However, investments are needed to strengthen trials’ research infrastructure,^66^, ^127^ scale up overall system capacity for the collection of PRO data and address implementation barriers and facilitators,^129^, ^130^ support method development,^131^ and create and sustain partnerships among trialists, PRO researchers, clinicians, regulators, and patient advocates,^127^, ^132^ particularly in the context of early‐phase clinical trials. Research is ongoing to evaluate alternative methods for AE reporting, including electronic PRO collection and alerts,^127^, ^133^ automated extraction from the electronic health record,^134^, ^135^ implementation of triggers and alerts to improve data quality,^49^ novel tolerability indicators that incorporate patient‐generated health data,^136^ and analytic methods to understand the longitudinal profile and cumulative impact of nonsymptomatic and symptomatic AEs rather than only the highest AE grade.^137^, ^138^ Efforts are also ongoing to examine interventions to improve health care professionals’ practices in reporting AEs.^139^

### Conclusions

In this commentary, we have outlined the key concepts to be considered when evaluating cancer treatment tolerability, describing direct and indirect indicators of tolerability and emphasizing the important role of PRO measures in a wide range of tolerability‐focused study designs. We have also highlighted the ways in which whole‐person factors meaningfully influence the generalizability of conclusions about tolerability, particularly with respect to understanding the experiences of distinct subgroups or the factors that can be addressed to improve the tolerability of cancer treatments.

Strengthening the measurement and interpretation of tolerability enhances our understanding of patient risk, benefit, and outcomes in both cancer treatment trials and comparative effectiveness research conducted in real‐world settings. A more precise and comprehensive measurement of tolerability is essential for shared decision‐making, and can inform the provision of tailored supportive care. To address the need to better integrate and analyze different measures of tolerability, the National Cancer Institute funded the Cancer Treatment Tolerability Consortium.^140^, ^141^ Ultimately, these insights can inform future clinical trials and advance the delivery of individualized cancer care.

## AUTHOR CONTRIBUTIONS

**Sandra A. Mitchell**: Conceptualization; writing—original draft; and writing—review and editing. **Rachel D. Altshuler**: Conceptualization; writing—original draft; and writing—review and editing. **Diane C. St. Germain**: Conceptualization; writing—original draft; and writing—review and editing. **Brennan Parmelee Streck**: Writing—original draft; conceptualization; and writing—review and editing. **Alice P. Chen**: Conceptualization; writing—original draft; and writing—review and editing. **Lori M. Minasian**: Conceptualization; writing—original draft; and writing—review and editing.

## CONFLICT OF INTEREST STATEMENT

Brennan Parmelee Streck reports holding stock with Gilead Sciences, Johnson & Johnson, and Pfizer. The other authors declare no conflicts of interest.

## Data Availability

The original contributions are included in the article; further inquiries can be directed to the corresponding author.
